# Correlation of Clinical Severity and Laboratory Parameters with Various Serotypes in Dengue Virus: A Hospital-Based Study

**DOI:** 10.1155/2020/6658445

**Published:** 2020-12-15

**Authors:** Pooja Rao, Achappa Basavaprabhu, Suchitra Shenoy, Nikhil Victor Dsouza, Basavaiah Sridevi Hanaganahalli, Vaman Kulkarni

**Affiliations:** ^1^Department of Microbiology, Kasturba Medical College, Mangalore, Manipal Academy of Higher Education, Manipal, Karnataka-575001, India; ^2^Manipal Center for Infectious Diseases, Prasanna School of Public Health, Manipal Academy of Higher Education, Manipal, Karnataka 576104, India; ^3^Department of Internal Medicine, Kasturba Medical College, Mangalore, Manipal Academy of Higher Education, Manipal, Karnataka-575001, India; ^4^Department of Pathology, Kasturba Medical College, Mangalore, Manipal Academy of Higher Education, Manipal, Karnataka-575001, India; ^5^Department of Community Medicine, Kasturba Medical College, Mangalore, Manipal Academy of Higher Education, Manipal, Karnataka-575001, India

## Abstract

**Objectives:**

Dengue fever, being hyperendemic with analogous presentations as in many other acute febrile illnesses, poses a challenge in diagnosis during the acute stage. Additionally, the coexistence of multiple serotypes further complicates the disease prognosis. The study was undertaken to determine the dengue virus serotypes, clinical, and laboratory markers as predictors in the severity of infection.

**Methods:**

A prospective study was conducted among 106 patients admitted with acute febrile illness having positive NS1 antigen/IgM ELISA. Clinical data were extracted from medical records including demographics, presence of comorbid conditions, clinical presentation, laboratory investigations, and course including length of hospital stay and outcome. Detection of dengue serotypes was done by multiplex reverse transcriptase polymerase chain reaction (RT_PCR).

**Results:**

Out of 106 RT-PCR-confirmed cases, DENV-3 was the most common serotype found in 56 (52.8%) patients, followed by DENV-3 and DENV-4 coinfection in 27 (25.4%) patients. Coinfection with more than one serotype was witnessed in our study. Raised liver enzymes and increased ferritin are good biomarkers in differentiating dengue from severe dengue with cutoff levels for AST (134 U/L), ALT (88 U/L), and ferritin (3670 ng/ml). Musculoskeletal, followed by gastrointestinal, manifestations were comparatively higher than respiratory and cutaneous manifestations.

**Conclusion:**

This study provides more information on the dengue serotypes. The clinical spectrum along with laboratory parameters such as ferritin, liver enzymes, platelet can be used as potential biomarkers in prediction of dengue severity. The data demonstrated will be useful in early detection and monitoring of the disease.

## 1. Introduction

Dengue fever (DF) is a major public health issue. It is a common mosquito-borne illness that is endemic in the coastal region of South India. Global burden estimate indicates 390 million infections per year. Among these, 70% actual burden was seen in Asia [1]. It is an important cause of mortality and morbidity among the vector-borne illness.

Four antigenically different dengue virus serotypes (DENV-1, DENV-2, DENV-3, and DENV-4) are known to cause infections in humans. Chances for developing dengue hemorrhagic fever-dengue shock syndrome (DHF-DSS) increases significantly with a history of the previous infection with one of the four serotypes. Early diagnosis, serotyping, and providing timely warning of dengue fever epidemics to the concerned authorities become very important for better patient outcomes and to curb the rapid spread of the virulent serotypes within the community [2]. The hyperendemicity with coinfection of two or more serotypes during the same period has been widely suspected as one of the major causes of disease severity in dengue patients in India [3]. Dengue surveillance becomes important when multiple serotypes are prevalent in one individual [4].

In the current study, we aimed (1) to identify the dengue virus serotypes in clinically suspected cases with dengue, as its distribution in this region is not documented, (2) to determine the different serotype-specific clinical manifestations and its association with disease severity, and (3) to study the association of markers such as ferritin, platelet count, and differential leucocyte count as risk predictors in assessing the severity of dengue.

## 2. Methods

A prospective study was conducted in patients admitted to the tertiary care center in Mangalore, India, with clinical suspected signs and symptoms of dengue during the dengue outbreak 2019 in Dakshina Kannada district of Karnataka. This region is endemic for dengue, and a steep rise in cases is seen during every rainy season. Patients above 18 years were included in the study. Cases that were treated on an outpatient basis or those who were less 18 years of age were excluded from this study.

Out of 3,801 suspected patients with fever during the period of July to October 2019, 991 were tested positive by NS1/IgM ELISA. Serotype analysis was performed for 106 patients. Patients were categorized based on WHO classification as stage 1—dengue without warning signs, stage 2—dengue with warning signs, and stage 3—severe dengue. A sample size of 106 was calculated considering a power of 80%, confidence level of 95%, proportion of DENV-1 to be 68.8%, and absolute precision of 5%.

The formula used is

(1) $$\begin{array}{c} N=\frac{4PQ}{D^{2}}, \end{array}$$

where *N* = sample size, *P* = proportion of interest (68.8%), *Q* = 1 − *P*, and *D* = absolute precision (5%) [3].

### 2.1. Sampling Technique: Convenient Sampling

#### 2.1.1. Serological Diagnosis

Acute phase serum samples within 7 days of onset of symptoms which were positive for dengue NS1 rapid immunochromatographic test/IgM positive by ELISA (Panbio) were analyzed from 106 patients. The samples were stored at −20°C.

#### 2.1.2. Molecular Diagnosis

The viral RNA was extracted using QIAamp Viral RNA Mini Extraction Kit (Qiagen, Germany) as per the kit protocol. The extracted RNA was stored at −80°C until use. The multiplex one-step reverse transcriptase PCR was carried out for dengue serotype confirmation. The CDC dengue primers and probes and the master mix One Step Prime Script™ RT-PCR Kit (Takara Bio, Japan) were utilized in this study. A multiplex assay was performed for detecting the 4 serotypes DENV-1, DENV-2, DENV-3, and DENV-4. The PCR amplification was carried out with thermal cycling conditions as per the CDC Dengue Kit protocol: hold at 30 min at 50°C, holding cycle 2 : 2.0 min at 95°C; florescence acquiring at 15 sec at 95°C and 1 min at 60.0°C for 45 cycles. The amplified products were accounted for an increase in fluorescence detection in a specific channel. The positive control was provided in the kit.

The study was conducted after the approval from the institutional ethics committee and with the informed consent from the patients.

### 2.2. Statistical Analysis

The categorical data were analyzed in the form of frequency and proportion. The quantitative data were analyzed in the form of mean, median, and proportion. The complete data were entered and analyzed in SPSS version 17. For the quantitative data, receptor operator curve (ROC) was plotted to establish the cutoff concentration. For ferritin, Kruskal–Wallis test was used to study the relation with severity in dengue. A *p* value <0.05 was considered as significant.

## 3. Results

**Figure 1:**
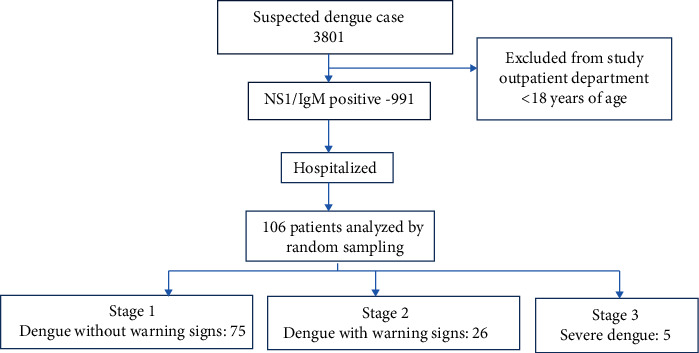
Flowchart depicting the case distribution.

Among 106 confirmed positive cases, dengue was predominantly seen in males (68) than in females (38) (Figure 1). According to age-group-wise distribution, more positive cases were seen in the age group of 21–30 years (36) followed by 31–40 years (29), 51–60 years (14), 18–20 years, 41–50 years (11), and above 61 years (5). The mean age was 35.27.

**Figure 2:**
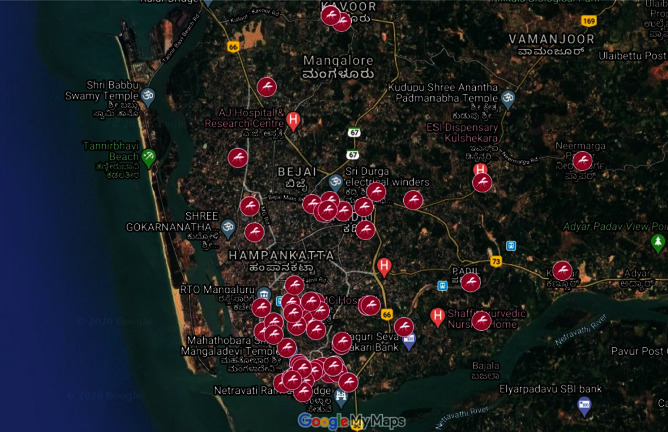
Geospatial mapping of positive dengue cases by RT-PCR in this region.

The incidence of different dengue serotypes in this region is shown in Figure 2.

The various comorbidities associated with dengue were diabetes mellitus (10.4%), diabetes with hypertension (0.9%), and malignancy (0.9%). According to WHO dengue classification, 70.8%, 24.5%, and 4.7% belonged to stages 1, 2, and 3, respectively.

All cases have presented with fever ranging from 99°C to 104.5°C followed by symptoms such as headache, malaise, chills, and rigors. Musculoskeletal manifestations such as myalgia and backache followed by gastrointestinal manifestations including vomiting, abdominal pain, and ascites were the next common clinical presentation, with the least number presenting with respiratory manifestations such as cough and cold and cutaneous manifestations such as rash and erythema. DENV-3 followed by mixed serotypes DENV-3 and DENV-4 presented with the above symptoms. Rash and cough was not a manifestation seen in the serotypes commonly found in the present study (Figure 1 and Table 1).

### 3.1. Laboratory Parameters

#### 3.1.1. Ferritin

In our study, the association of ferritin levels in patients categorized according to WHO classification of dengue was significant with a *p* value of 0.009 and interquartile range from 925 ng/ml–13,829 ng/ml. The maximum ferritin level rise being 87,945 ng/ml was noticed in a severe dengue case. The mean ferritin levels in dengue without warning signs, dengue with warning signs, and severe dengue were 2,304.48, 12,431.00, and 54655.25 ng/ml, and the cutoff values were 640, 2458, and 35,930 ng/ml, respectively. The cutoff for ferritin to differentiate between dengue and severe dengue was 3670 ng/ml. The area under the curve (AUC) for ferritin obtained in predicting dengue versus severe dengue is 0.849 with 95% CI (0.712, 0.985).

#### 3.1.2. Platelet Levels

The lowest platelet level observed during the stay in the hospital in stage 1 was 3000, stage 2 was 46,076, and stage 3 was 58,400 cells per mm^3^. The median platelet count in stage 1 was 85,000, stage 2 was 31,500, and stage 3 was 33,000. The *p* value was significant with <0.05. The AUC for maximum platelet drop obtained in predicting dengue versus severe dengue is 0.635 with 95% CI (0.454, 0.817). IQR in stage 1 was 53,000–85,000, stage 2 was 16,250–31,500, and stage 3 was 22,000–33,000. The maximum platelet drop during the stay in the hospital did not show any significance as a predictor of dengue severity. A total of 9 patients had platelet count above 1,50,000 cells/mm^3^.

#### 3.1.3. Liver Enzymes

**Figure 3:**
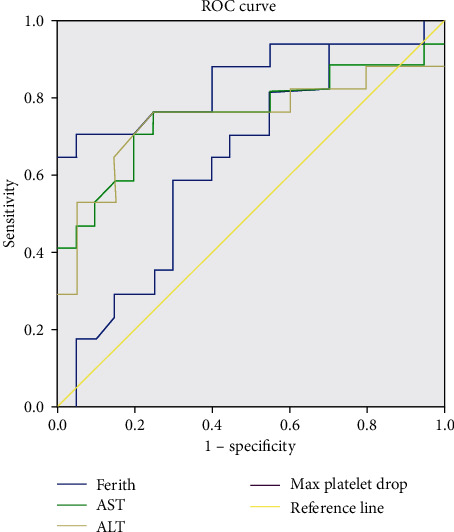
ROC curve for various biomarkers as predictors of dengue severity.

Serum ALT and AST observed were in the higher range with a maximum value of ALT and AST being 502 (IQR, 28–153 U/L) and 1737 (IQR, 40–240 U/L), respectively. On plotting the receiver operating characteristics (ROC), the cutoff for AST was 134 U/L and ALT was 88 U/L. The AUC for AST and ALT obtained in predicting dengue versus severe dengue is 0.757, 0.731 with 95% CI (0.586, 0.928) and (0.551, 0.911) (Figure 3).

An association USG gall bladder with the severity of dengue was significant (*p* value 0.001). The fatty liver change was a common feature observed in our study. Hemophagocytic lymphohistiocytosis (HLH) syndrome was seen in 8.5% of infected individuals (Table 2).

### 3.2. Serotype Analysis

The most common monotypic serotype in this region was DENV-3 seen in 56 (52.8%) patients, followed by multiple serotypes DENV-3 and 4 in 27 (25.4%) patients; all 4 serotypes in 8 (7.5%) patients; and DENV-2, 3, and 4 in 7 (6.6%) of patients (Table 3). Multiple serotypes, with more than one type, were a feature, and DENV-3 was a common serotype in these cases. The serotype distribution pattern was DENV-3 serotype and was found in 93 patients, DENV-4 in 39 patients, and DENV-2 in 21 patients followed by DENV-1 in 12 patients (Table 3).

No association was found between the severity of disease and serotype, as the percentage of severe dengue was less in number. DENV-3 was found in 8 out of 9 cases of HLH syndrome. The least common serotype was DENV-1. No fatalities were observed in the patients included in this study.

## 4. Discussion

Dengue fever has a dynamic pattern ranging from mild febrile illness to a spectrum of manifestations including hemorrhage, multiorgan dysfunction, HLH, and death. In the present study, the pattern of the clinical and laboratory parameter in patients during the monsoon season 2019 dengue outbreak in Dakshina Kannada was studied. With 991 dengue IgM confirmed cases during four months, dengue continues to be an endemic disease. The male population has dengue predominately in the ratio of 2 : 1. In other studies, a similar male preponderance was seen [4]. The probability is because this region is endemic for dengue and exposure of males to *Aedes* mosquito at their work place or while traveling. In our population, dengue was seen in age group between 20 and 40 years which could be attributed to increased movement of adults for earning their livelihood, easy accessibility to health care facilities, and awareness about high prevalence of mosquito-borne illness in the area.

On clinical evaluation, WHO stage 1-type DF was found in a higher number of cases than severe dengue of stage 2 and stage 3, which was comparable with studies conducted in Bali [5]. All four serotypes existed during the study period. The data show the most common serotype prevalent in this geographical region is DENV-3 which was seen in a study in Indonesia and in different parts of India such as Kerala and Uttar Pradesh [4, 6]. In Delhi, the DENV-2 serotype was predominant [7]. There was no association of serotypes with the severity of the disease. There is considerable variation in virulence of infecting serotypes which is dynamic. A comparative analysis of molecular serotyping could not be made as ours was the first study on dengue serotyping in this region. Studies have shown the coexistence of multiple serotypes in a single patient as a contributing factor in increased severity of dengue in these cases [8, 9].

High prevalence of gastrointestinal and musculoskeletal symptoms was seen in DENV-3 in a study conducted in Malaysia and India which is similar to our study, and cutaneous and respiratory symptoms were observed in DENV-4 which altered as DENV-4 was not prevalent in the current study [8, 10]. In a study conducted in the Taiwan population, DENV-3 cases presented with rash compared to DENV-2 [11]. Another study by Kumaria showed DENV-4 presenting with hemorrhagic manifestations, but with low positivity DENV-4 rate [6]. A steady rise in the ferritin levels along with levels of 2,304 ng/ml in stage 1 DF and the highest level up to 87,945 ng/ml seen among severe dengue with HLH syndrome correlated well with studies conducted by Roy Chaudhuri et al. and Soundaravally et al. [12, 13]. Thus, ferritin can be an important serological biomarker in the diagnosis and prognosis of DF. Total cell count was diminished in DF but did not draw parallel with the severity index. The liver enzymes such as serum ALT and AST were significantly increased asserting that these parameters could also be used as markers for prediction of dengue in acute febrile illness cases. Hyperferritinemia along with raised ALT and AST levels were noted as stated in a study by Ho et al. [14]. Increase in ALT and AST levels were seen as a marker to differentiate from other AFI excluding other causes such as liver abscess and acute hepatitis [15]. Thrombocytopenia was a feature in most of the cases with DENV-3 in our study which was similar to a study by Tsai et al. [11].

## 5. Conclusion

DENV-3 serotype and DENV-3 and DENV-4 serotype coinfection were prevalent in the Dakshina Kannada region of Karnataka, India. The present study concludes that coinfections with more than one serotype were not associated with disease severity in dengue infection. The study also showed that biomarkers such as ferritin and serum AST and ALT can be better predictors to assess the disease severity, and serial estimation of these markers should be considered. These outcomes provide novel data of clinical and serotype characteristics for better management of dengue in the population.

## Figures and Tables

**Table 1:** Clinical and laboratory profile in dengue infection.

	Present (%)	Absent (%)
*Symptoms*		
Fever	100	NIL
Headache	58.50	41.5
Vomiting	39.6	60.4
Myalgia	55.7	44.3
Chills/rigors	51.9	48.1
Backache	23.6	76
Rash	7	99
Cough/cold	7.5	98.5
Chest pain/palpitations	NIL	100
Giddiness	13.2	86.8
Abdominal pain	10.4	93.4
Postural drop	5.7	99
Loose stools	3.8	96.2

*Signs*		
Decreased Urine output	18	98.1
Ascites	13.2	86.2
Bleeding	9	99

*Lab Parameters*		
Cytopenia	56	44
Thrombocytosis	9	99.1
Thrombocytopenia	99.1	9
ALT	85	21
AST	84	22

**Table 2:** Ultrasonography abdomen findings in dengue.

Ultrasound	Dengue classification		
	Dengue without warning signs (75)	Dengue with warning signs (26)	Severe dengue (5)
Fatty liver	68 (79.1%)	14 (16.3%)	4 (4.7%)
Gall bladder stones	2 (25.0%)	5 (62.5%)	1 (12.5%)
Gall bladder wall thickening	3 (42.9%)	4 (57.1%)	0
Splenomegaly	6 (7.9%)	8 (32.6%)	3 (60%)
Hepatomegaly	14 (16.3%)	5 (62.5%)	0

**Table 3:** Distribution of dengue serotype.

Serotype	Number
DENV-3	56
DENV-3 and DENV-4	27
DENV-2, 3, and 4	08
DENV-2 and DENV-3	04
All 4 serotypes	08
DENV-4	02
DENV-1	01

## Data Availability

The data sets used to support the findings of this study are available from the corresponding author upon request.
